# Nominal Group Technique consultation of a Pulmonary Rehabilitation Programme

**DOI:** 10.12688/f1000research.3-42.v1

**Published:** 2014-02-13

**Authors:** Hayley A Hutchings, Frances L Rapport, Sarah Wright, Marcus A Doel, Clare Clement, Keir E Lewis

**Affiliations:** 1 Patient and Population Health and Informatics Research (PPHI), College of Medicine, Institute of Life Sciences 2, Swansea University, Swansea, SA2 8PP, UK; 2 Centre for Urban Theory, Department of Geography, Swansea University, Swansea, SA2 8PP, UK

## Abstract

**Objective: **The purpose of the study was to determine what patients, professionals and significant others regarded as the most important positive- and challenging aspects of Pulmonary Rehabilitation Programmes for patients with Chronic Obstructive Pulmonary Disease (COPD) and to gain insight into how such programmes could be developed and improved.

**Method:** A modified Nominal Group Technique method was used in three consultation workshops (one with COPD patients who had recently undertaken a Pulmonary Rehabilitation Programme; one with ‘significant others’ of the same patients; one with secondary care professionals who deliver the Pulmonary Rehabilitation Programme).

**Results:** Each of three workshops resulted in the production of approximately ten positive- and ten challenging aspects related to Pulmonary Rehabilitation Programmes.  These were further developed by a process of thematisation into seven broad themes.  The most important was ‘ *the patient*’, followed by ‘ *physical health*’; jointly ranked as third were: ‘ *mental health*’ and ‘ *knowledge and education*’.  ‘ *The programme*’ and ‘ *professional characteristics*’ were jointly ranked as fifth, with ‘ *the future*’ being ranked as the least important theme.

**Conclusions:** The modified Nominal Group Technique method allowed the development of a ranked thematic list that illustrated the important positive- and challenging aspects of Pulmonary Rehabilitation Programmes for patients with COPD. These themes should be core to planning future Pulmonary Rehabilitation Programmes, particularly if patients and carer views are to be considered.

## Introduction

Chronic Obstructive Pulmonary Disease (COPD) is a progressively disabling condition characterised by impaired respiratory function associated with physical limitations and psychological co-morbidity ^1^. COPD results in a reduced capacity for functional activities and performance of daily activities with a corresponding impairment in Health Related Quality of Life ^2^. Current figures show 900,000 people have been diagnosed with and are receiving treatment for COPD within the United Kingdom ^3^. However, due to under reporting or under diagnosis, the actual number of those suffering with COPD is estimated to be as high as 3 million ^4^. Stopping smoking is crucial and is the only intervention that influences the natural history of lung deterioration, with current pharmacological treatment being aimed at reducing symptoms and exacerbations ^5^.

Pulmonary Rehabilitation Programmes are multi-disciplinary interventions individually tailored to optimise each patient’s physical and social performance. Rigorous evidence from randomised controlled trials demonstrates that Pulmonary Rehabilitation Programmes for COPD can improve dyspnoea, exercise tolerance, Health Related Quality of Life, and reduce the number of days spent in hospital and the utilisation of healthcare resources ^6– 8^. Pulmonary Rehabilitation Programmes have been shown to be cost-effective and are now recommended for all patients who remain breathless despite optimal bronchodilators, irrespective of severity and age ^6– 9^. Pulmonary Rehabilitation Programmes are also being effectively applied to non-COPD causes of pulmonary impairment ^10^.

There are now specific guidelines and recommendations in the United Kingdom regarding Pulmonary Rehabilitation Programmes, including how to select patients, the timing and number of sessions, intensity and type of exercise, the key educational, psychological and behavioural components, oxygen supplementation and outcome assessment ^7, 8^. Research exploring the benefits following Pulmonary Rehabilitation Programmes has predominantly been quantitative in nature. There have been some qualitative studies with COPD patients, but these have focused largely on specific aspects of patient experience ^11, 12^ and barriers to participation in Pulmonary Rehabilitation Programmes or other self-management programmes ^13, 14^. There has been some exploration of the effectiveness of self-management programmes from the patient perspective ^15– 17^. However, none of these studies have combined patient, carer, and professional perspectives, particularly in an in-depth analysis regarding the long-term impact of Pulmonary Rehabilitation Programmes in relation to personal needs and issues such as perceived patient benefits, and expectations and challenges of Pulmonary Rehabilitation Programmes. It has been recognised that a better understanding of how Pulmonary Rehabilitation Programmes improve Health Related Quality of Life could affect the future design of programmes, enhance measurement tools for Health Related Quality of Life and more appropriately support the specific needs of patients ^15, 17, 18^.

Consensus methods are techniques used gain opinions and views from appropriate experts regarding the current position in a particular field. They provide a mechanism for assimilating and synthesising information, particularly where published information may be inadequate or non-existent ^19^. The purpose of consensus methods is to reach an agreement on a particular issue. Consensus methods can also mitigate some of the problems sometimes associated with group decision-making processes. In particular, where dominant views may lead the process and crowd out other perspectives ^19^.

Nominal Group Technique is one of the commonly used consensus methods within healthcare and medical settings. The technique was first developed as an organisational planning technique by Delbecq *et al.* in the 1970s ^20^. The Nominal Group Technique normally involves four main phases: a nominal phase, during which each individual silently considers the issues under deliberation; an item-generation phase, during which each individual discloses the results of their deliberation to the group; a discussion and clarification phase, during which the group assures itself that it has understood the items that have been advanced; and a voting phase, during which the items are evaluated and the issue is decided (e.g. a ranking exercise). Nominal Group Technique promotes individual contributions allowing each individual the opportunity to voice their opinions. Factors that would normally inhibit participation are therefore avoided and even the more reticent group members are encouraged to participate in all phases ^21^.

By adopting a mixed methods design, employing qualitative and quantitative methods during consultation with mixed stakeholder groups, and by including a modified Nominal Group Technique component as described previously ^22^, we aimed to provide a picture of the perceived benefits and challenges of Pulmonary Rehabilitation Programmes for COPD patients. Here we report the quantitative analysis of the Nominal Group Technique activities.

## Methods

Following regional ethics and research and development approval, a series of consultation workshops were held between January and December 2012, in a District General Hospital in Wales, United Kingdom, serving a mixture of urban and agricultural communities. The hospital delivers a regular Pulmonary Rehabilitation Programme which includes 18 sessions of outpatient multidisciplinary input from occupational therapists, physiotherapists, dietetics staff, physicians, specialist respiratory nurses, social workers and a smoking cessation counsellor. The content and timings of the Pulmonary Rehabilitation Programme is evidenced-based and is tailored to individual requirements and personalised goal setting.

### Participants

We recruited across the South West Wales Regional Health Board, United Kingdom that serves 385,000 people and included patient, professional and significant other groups, to ensure we included a wide range of views, experience and knowledge of COPD and Pulmonary Rehabilitation Programmes.

Patients with COPD who were currently participating in or who had completed a Pulmonary Rehabilitation Programme within the last 2 years were approached to participate in the study, with most being approached in their last Pulmonary Rehabilitation Programme session. Information sheets were given to patients for their significant others (husbands, wives, partners, friends, carers or family members) inviting them to contact the researcher if they wished to participate. Professionals who were identified as playing a significant role in the delivery of the Pulmonary Rehabilitation Programmes and the treatment of COPD patients (occupational therapists, physiotherapists, respiratory consultants, respiratory team administrators, pharmacists, counsellors, psychologists, and specialist respiratory nurses) were also approached to participate in the study. All 20 participants (8 patients, 8 professionals and 4 significant others) provided written informed consent.

### Study design

Our aim was to gain an understanding of the positive and challenging aspects of Pulmonary Rehabilitation Programmes for patients with COPD and to gain a consensus regarding what constitute the most important aspects of Pulmonary Rehabilitation Programmes.

### Consultation workshops

Nominal Group Technique consensus exercises were carried out as one aspect of a multi-layered, mixed-method consultation during three half-day workshops (one with professionals, one with COPD patients, and one with the significant others of patients). Based on guidance in the literature for optimal numbers for qualitative group consultations, we aimed to recruit six participants to each of the three workshops ^23^.

**Figure 1.**
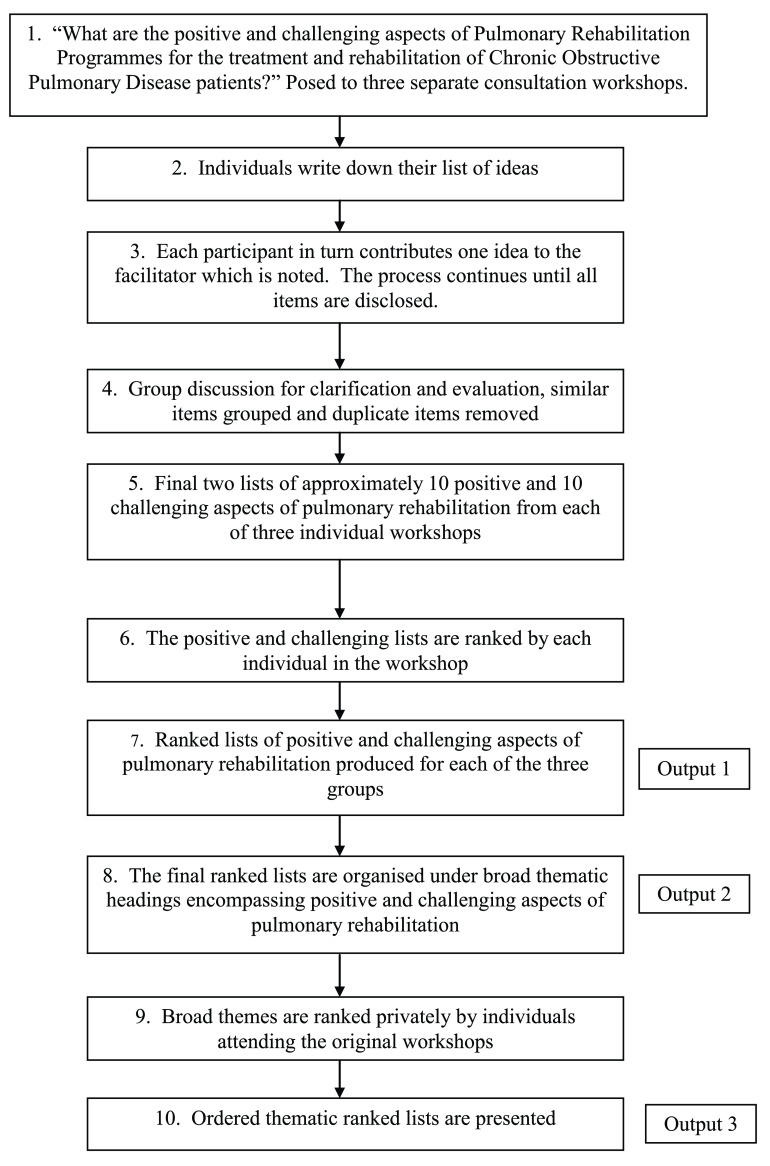
Flow diagram steps involved in the Nominal Group Technique process of the study.

Each workshop was made up of three parts. Part one began with a broad discussion that examined the nature and content of Pulmonary Rehabilitation Programmes through a semi-structured group interview. The second part involved more extensive discussion with participants. Having attended a Pulmonary Rehabilitation Programme, participants were encouraged, using personal examples to describe what the Programme meant to them. This included exploring their perceived views regarding the benefits and challenges of Pulmonary Rehabilitation Programmes and impact on patient Health Related Quality of Life. An adapted Nominal Group Technique exercise was employed in the final part of the workshop. The focus of this stage was to address the following question with participants: “what are the positive, and what are the challenging aspects of Pulmonary Rehabilitation Programmes for the treatment and rehabilitation of COPD patients?” During the Nominal Group Technique exercise, issues that were raised in the early parts of the workshop were refined and condensed into a list of approximately ten positive and ten challenging aspects. At the end of the workshop, participants were asked to rank these aspects in order of significance (Steps 1–7, leading to Output 1, Figure 1). The generation of the positive and challenging aspects of the Pulmonary Rehabilitation Programme using Nominal Group Technique followed the standard approach outlined in previous work ^22^.

The data generated from each Nominal Group Technique activity (Output 1, Figure 1) were collated for each consultation workshop. Median ranks with interquartile ranges were calculated using SPSS version 19 for each of the aspects on the positive and challenging lists and a consensus ranked list was produced based on these final median ranks.

Nominal Group Technique consultation of a Pulmonary Rehabilitation Programme Data Set Dataset 1: NGT activity with patients- positive aspects Final agreed list of positive aspects of a Pulmonary Rehabilitation Programme for COPD patients. Data are illustrated for each of the patient participants and the generated aspects are ranked in order of importance according to their calculated median and inter-quartile range (IQR) scores. Dataset 2: NGT activity with patients- challenging aspects Final agreed list of challenging aspects of a Pulmonary Rehabilitation Programme for COPD patients. Data are illustrated for each of the patient participants and the generated aspects are ranked in order of importance according to their calculated median and inter-quartile range (IQR) scores. Dataset 3: NGT activity with significant others- positive aspects Final agreed list of positive aspects of a Pulmonary Rehabilitation Programme for COPD patients. Data are illustrated for each of the significant other (Sig oth) participants and the generated aspects are ranked in order of importance according to their calculated median and inter-quartile range (IQR) scores. Dataset 4: NGT activity with significant others- challenging aspects Final agreed list of challenging aspects of a Pulmonary Rehabilitation Programme for COPD patients. Data are illustrated for each of the significant other (Sig oth) participants and the generated aspects are ranked in order of importance according to their calculated median and inter-quartile range (IQR) scores. Dataset 5: NGT activity with professionals- positive aspects Final agreed list of positive aspects of a Pulmonary Rehabilitation Programme for COPD patients. Data are illustrated for each of the professional (Prof) participants and the generated aspects are ranked in order of importance according to their calculated median and inter-quartile range (IQR) scores. Dataset 6: NGT activity with professions- challenging aspects Final agreed list of challenging aspects of a Pulmonary Rehabilitation Programme for COPD patients. Data are illustrated for each of the professional (Prof) participants and the generated aspects are ranked in order of importance according to their calculated median and inter-quartile range (IQR) scores. Dataset 7: Thematic NGT activity Final agreed thematic consensus list of positive and challenging aspects of a Pulmonary Rehabilitation Programme for COPD patients. Data are illustrated for all the participants and the generated aspects are ranked in order of importance according to their calculated median and inter-quartile range (IQR) scores.

### Generation of themes

Following the consultation workshops we adapted the Nominal Group Technique method as previously described ^22^ in order to include an additional multi-group ranking round (Steps 8–10, Figure 1). The lists of positive and challenging aspects of a Pulmonary Rehabilitation Programme produced following the three workshops were organised into a series of over-arching themes under which the positive and challenging aspects fitted (Step 8, Output 2, Figure 1). Rigour was maintained throughout the process of theme generation, by adhering to recommended qualitative data reliability and validity techniques ^24– 26^. An independent analysis of the lists generated from the workshops was carried out by two of the study team in order to identify the key over-arching themes. This process involved deletion of duplicate items and amalgamation of items where overlap was clear. A final set of common themes was independently generated by a third member of the team. This reflected and amalgamated the thematisations of the first two.

### Thematic consensus

Following the generation of themes, all the original workshop participants were sent a pack of A5-sized cards. Each card carried a broad theme as a header under which were listed the associated set of positive and challenging aspects. As with the earlier workshop Nominal Group Technique activity, participants were asked to rank the themes in order of importance: with ‘1’ representing the theme they regarded as being most important and subsequent ranks signifying the themes of diminishing importance (Step 9, Figure 1) ^22^. The ranked cards were returned by participants in a pre-paid envelope.

The data from the returned cards were analysed using SPSS version 19 in order to calculate the median ranks and interquartile ranges (IQR) for each of the themes. A final consensus ranked thematic list was produced based on these median ranks (Step 10, Figure 1). This was the list produced for discussion and dissemination ensuring veracity within the method and enabling cross-consideration of themes and aspects by team members from Stage 1 thematisation undertaken within a group setting, to Stage 2 thematisation, undertaken by individual participants, post-consultation workshop.

### Thematic template generation

Notes and audio recordings from the three consultation workshops were transcribed. These transcripts were subjected to thematic and summative analysis to extract relevant information related to each of the generated themes ^27, 28^. The detailed content relating to each theme was extracted from the individual transcripts and was built up to articulate fully the set of aspects that it contained and to clarify any anomalies or ambiguities ^29^. The final output of the consultation workshop was a ‘thematic template’ that ranked each theme in order and that provided a qualitative in-depth elaboration of the content contained within each theme.

## Results

### Consultation workshops

We recruited a total of 20 participants across the three consultation workshops (see Table 1). Thirty three positive and 35 challenging aspects of Pulmonary Rehabilitation Programmes were produced in total for the three workshop group. The ranked list for each of the consultation workshops is illustrated in Table 2.

**Table 1.** Summary of three Chronic Obstructive Pulmonary Disease (COPD) workshop participant samples.

	Study group	Male/female	Participant status	Age	Date of PR programme	Year of diagnosis
1	SO	M	Significant other	n/a	n/a	n/a
2	SO	F	Significant other	n/a	n/a	n/a
3	SO	F	Significant other	n/a	n/a	n/a
4	SO	F	Significant other	n/a	n/a	n/a
5	PROF	M	Consultant respiratory physician	n/a	n/a	n/a
6	PROF	M	Consultant respiratory physician	n/a	n/a	n/a
7	PROF	M	Pharmacist	n/a	n/a	n/a
8	PROF	F	Occupational therapist	n/a	n/a	n/a
9	PROF	F	Specialist respiratory nurse	n/a	n/a	n/a
10	PROF	F	Physiotherapist	n/a	n/a	n/a
11	PROF	F	Administrator	n/a	n/a	n/a
12	PROF	F	Dietician	n/a	n/a	n/a
13	PT	M	COPD Patient	73	2005	2005
14	PT	M	COPD Patient	66	2010	2010
15	PT	F	COPD Patient	54	2011	2011
16	PT	M	COPD Patient	62	2011	2011
17	PT	M	COPD Patient	66	2011	2009
18	PT	M	COPD Patient	72	2011	1998
19	PT	F	COPD Patient	69	2012	2005
20	PT	M	COPD Patient	74	2012	2005

SO, significant others; PROF, professionals; PT, patients; n/a, not available.

**Table 2.** Positive and challenging aspects generated by each of the study workshops.

Group	Positive aspects (n=33)	Challenging aspects (n=35)
**Patients**	1. Breathing properly	1. Lack of privacy (corridor walking test)
	2. Breaking the cycle of inactivity	2. Poor communication between clinicians
	3. Relaxation	3. Venue not ideal (physiotherapy gym)
	4. Self-help; awareness; empowerment	4. Lack of funding
	5. Physical benefits	5. Explanation why there is a delay/need to wait
	6. Mental strength	6. Daunting experience at the outset
	7. Knowledge	7. Lack of clarity about what the programme is about
	8. Control panic attacks	8. Diet information (one-sided: weight gain)
	9. Legacy of the future (hopes, lasting change)	9. Commitment-insufficient for programme
	10. Morale, self-esteem, feel-good factor	10. Waiting (to get on the programme)
		11. Poor state of information from GPs
**Professionals**	1. Patient improvement	1. Waiting-time lists
	2. Life enhancement	2. Capacity/space constraints
	3. Patient improved attitude to condition	3. Lack of flexibility to run in other locations
	4. Graduated exercise	4. Time wasters/patients who do not attend
	5. Multi-disciplinary team approach	5. Drop-out rate high
	6. Patient education/demystification/knowledge	6. Travel and financial constraints
	7. Complementary/holistic - more than just a pill	7. Convincing patients of benefits
	8. Good evidence base	8. Lack of staff resources
	9. Validation of anxiety and confidence	9. Lack of time to improve programme
	10. Patient satisfaction/appreciation of service	10. Inability to sufficiently individualise programme
	11. Staff reward and motivation	11. Long term benefits still unknown
		12. Lack of follow-up
**Significant** **Others**	1. Time for yourself	1. Coming for the first time
	2. Partner’s enthusiasm and enjoyment	2. Uncertainty about what to expect
	3. A learning experience	3. Challenging activities
	4. Gaining confidence	4. Personal motivation to keep going
	5. Knowing help was available	5. Lack of funding
	6. Caring staff	6. Not knowing the bigger picture
	7. Given sufficient time	7. Being over-protective
	8. Friendships made	8. Learning not to take over
	9. Learning to manage illness	9. Poor relationships with GPs and staff
	10. Physical and mental improvement and independence	10. Lack of GP and staff knowledge
	11. Programme sustained	11. Worsening of the condition in the longer term
	12. Saving money for the health services	12. No opportunity for future follow-ups

The positive and challenging aspects within each workshop group list represent the ranked lists ordered by the individuals in each group. The aspects generated are based on direct quotes from the individuals attending the workshops.

### Generation of themes

Individual assimilation produced similar lists of common broad themes that were refined to seven (Output 2, Figure 1). The seven themes were: *the patient*, *physical health*, *mental health*, *knowledge and education*, *the programme*, *professionals and significant others* and *the future* (see Table 3).

**Table 3.** Final themes encompassing positive and challenging aspects of Pulmonary Rehabilitation Programmes.

Theme	Positive aspects	Negative aspects
The Patient	Patients gain an improved awareness and appreciation of their condition Patients gain confidence from attending Programme Programme supports self-help and empowerment for patient Programme helps patients recover aspects of everyday life Patient has enjoyed the experience	Daunting experience at outset and attending Programme for the first time Insufficient commitment to Programme Time wasters/‘Did Not Attend’ (DNAs) Challenges physical ability of patients Lack of personal motivation to keep going
Physical Health	Being able to breathe properly Control anxiety and panic through relaxation Family and friends using same physical techniques for themselves Patient experiencing improvements in own health Developing more independence as result of physical improvements Breaking the habit of feeling physically inadequate	Physical activity
Mental Health	Gaining an improved attitude to the condition Developing mental strength and confidence Ability to live a more fulfilling life Experiencing a ‘feel-good’ factor and increased sense of self esteem Perceiving improvements in one’s health Better state of mind	To keep motivated and ‘keep going’ Controlling panic attacks
The Programme	Programme saves health service money Programme is complementary and holistic in approach – far more than a ‘pill’ Programme should be sustained Gradual increase in exercise across the Programme Good scientific evidence that the Programme works Patient and partner satisfaction with Programme Multi-disciplinary approach during Programme sessions Programme provides time and independence for significant other Appropriate duration and frequency of Programme sessions	Uncertainty about what the Programme entails Attending Programme for the first time Funding for Programme Patient travel and financial constraints Capacity and space for Programme Limitations to running Programme in small number of locations Programme is not individualized enough Lack of privacy Inappropriately shared professional, public and patient spaces (e.g. professionals eating lunch in gym) High drop-out rate Time wasters/‘Did Not Attend’ (DNAs) Lengthy waiting lists Lack of time to improve Programme
Professionals and Significant Others	Friendships made Partner’s enthusiasm and enjoyment Multi-disciplinary, professional team with good-skill mix Caring staff Motivated staff Staff feeling rewarded by the Programme work Programme provides time and independence for significant other Patients know that help is available	Lack of staff resource Poor relationships with GPs and other staff Poor communication between clinicians and between Trusts Convincing patients of benefits of Programme Explaining delays to patients of getting on Programme Significant others being over-protective of patients Significant others learning not to take over
The Future	Positive legacy of Programme	Structured follow-up is not offered Worsening of condition in the future Long-term benefits still unknown Lack of funding Sustainability of Programme Post–Programme assessment is not conducted at one year
Knowledge and Education	Programme provides knowledge and patient education Good scientific evidence for running the Programme Provides a learning experience for all concerned Knowledge and information helps individuals to manage their illness Demystifies the condition Being taught how to breathe properly Learning how to relax Patients passing on knowledge and skills gained from Programme to others	Lack of clarity at outset regarding what the Programme entails Lack of General Practice staff knowledge about Programme to support patients More dietary information required about weight loss rather than just weight gain Lack of clarity at outset of benefits of the Programme Not being fully informed about the potential delays in starting Programme

The positive and challenging aspects encompassed within each theme are based on direct quotes from the individuals attending the workshops.

### Thematic consensus

Fourteen of the 20 attendees at the three workshops returned the packs of cards. Two were incorrectly completed, resulting in 12 evaluable responses (60%).

Following thematic ranking, the theme that was regarded as most important was *the patient*, followed by *physical health*. Jointly ranked as third were: *mental health* and *knowledge and education*. *The programme* and *professionals and significant others* were jointly ranked as fifth, with *the future* ranked as the least important theme ( Table 4).

**Table 4.** Final ranked thematic list (n=12*).

Ordered rank (1–7)	Theme	Median rank (Interquartile range)
1	The Patient	1 (0)
2	Physical Health	2 (1)
=3	Mental Health	4 (1)
=3	Knowledge and Education	4 (2.5)
=5	The Programme	5 (1.75)
=5	Professionals and Significant Others	5 (2.25)
7	The Future	7 (1)

*Based on 12 evaluable responses. Total respondents=14 (14/20=70%), 2 were excluded from analysis due to incorrect completion.

### Thematic template generation

In summary, *the patient* detailed how the patient’s health and wellbeing changed for the better over the course of Pulmonary Rehabilitation, and how patients were encouraged to gain confidence, to demonstrate a commitment to improving their own health, and to adopt a broader outlook on ongoing healthcare needs and expectations. *Physical health* illustrated how learning to breathe “properly” had a profound impact on patients, not only because breathing well is vitally important to their health and quality of life, but also because breathing “properly” is something that needs to be learnt. *Mental health* highlighted that bringing patients together enabled them to appreciate that they were not alone in their feelings and experiences. *Knowledge and education* emphasised the ability of Pulmonary Rehabilitation Programmes to create a learning environment, lasting for many weeks, within which patients are educated about their illness, and are able to develop new techniques to manage and cope. In *the programme*, patients, professionals, and significant others all emphasised positive outcomes for patients attending Pulmonary Rehabilitation Programmes for the duration and in the longer-term: physically, mentally, and socially. *Professionals and significant others* discussed how patients regarded the professionals as “caring” and “friendly”, treating them with “dignity” and “respect”, and that this created a welcoming and safe environment that enabled them to feel “cared for” and “at ease”. With respect to the theme of *the future*, participants emphasised a plethora of benefits that could be directly attributed to Pulmonary Rehabilitation Programmes, including improved health outcomes, enhanced quality of life, fewer hospital admissions, less time spent in hospital and consequently health care financial savings.

## Discussion

We identified important aspects of Pulmonary Rehabilitation Programmes for the treatment of COPD from the point of view of a mixed population group of patients, professionals and significant others. Using a modified Nominal Group Technique exercise delivered during innovative consultation workshops, we produced a novel ranked thematic list that encompassed the important positive but also challenging aspects of Pulmonary Rehabilitation Programmes.

There was a surprisingly diverse range of generated aspects ( Table 2) across the three workshops. The professional outputs were focused on pragmatic service delivery, with a clear goal of patient improvement, education and attitudinal change. The patients focused not only on physical improvements but also on improving mental strength, morale and self-esteem. Although all patients were positive about Pulmonary Rehabilitation Programmes, they also highlighted the challenges faced by some of them in attending them, which included an occasional lack of privacy, instances of poor communication, inadequate venues for certain activities (e.g. a public area of a hospital corridor to perform shuttle walk tests) and being daunted by the prospect of exercise and gym work. These findings are in accord with previous literature, which has examined the reasons for non-attendance on Pulmonary Rehabilitation Programmes ^13, 17^. Interestingly, the significant others focused on the social elements, with friendships made, caring staff and individual care contributing to the patients’ gaining confidence and learning about how to manage their condition. The significant others also highlighted the knock-on-effect of allowing them to have more time for themselves and not be so protective of the patients. All participants recognised that they were unsure what the future would bring in terms of long-term health and health-care support, but were keen for continued contact with professionals, Pulmonary Rehabilitation Programmes refresher courses and for the Pulmonary Rehabilitation Programmes to be recognised as beneficial for others, and thus maintained.

The final outcome of the Nominal Group Technique exercise was a ranked list of seven themes ( Table 3), with ‘the patient’ ranked as the most important theme, followed by ‘physical health’. Overall, the main positive benefits of Pulmonary Rehabilitation Programmes were that they instilled confidence, enabled patients to breathe properly and manage their health more efficiently, encouraged the patient to be more self-sufficient and in control, and were enjoyable. The challenges to participation were that Pulmonary Rehabilitation Programmes were daunting, physically challenging, and required motivation. Interestingly, many of these challenges have been highlighted in previous qualitative studies ^16^ with COPD patients as important reasons why patients decline entry or withdraw from Pulmonary Rehabilitation Programmes. Patient beliefs about Pulmonary Rehabilitation Programmes can comprise positive aspects (e.g. that they will lead to improvement, safe and multi-disciplinary setting, and motivation) as well as negative aspects (they lead to disruption of normal routine, being tired, transport issues and limited privacy) ^13^. It has been shown that attending a Pulmonary Rehabilitation Programme is associated with better management of breathlessness, which in turn has a positive impact on physical and social activity, coping strategies and patient confidence ^15, 17, 18^.

This study was carried out within one geographical location in South-West Wales, United Kingdom, and employed only three consultation workshops. Whilst we are confident that the methods adopted are transferable, in line with our extensive engagement with the methods in a range of community and primary care settings ^22, 30^, a larger study, employing more consultation workshops conducted over a larger geographical area is necessary to consider whether all the important aspects of Pulmonary Rehabilitation Programmes have been revealed, and whether the themes we identified within this study are generalisable.

The adapted Nominal Group Technique exercise was a mechanism for distilling the important aspects of Pulmonary Rehabilitation Programmes in a mixed group of individuals, which allowed the views of all the participating groups to be considered as equal. The process of qualitative elaboration of these themes in terms of what they meant to patients, professionals and significant others, provided a more comprehensive picture than other studies have derived. Moreover, combining qualitative with quantitative assessments provides more information, and these approaches could be used to make recommendations to improve and develop Pulmonary Rehabilitation Programmes across health-care contexts.

## Data availability

*Figshare*: Nominal Group Technique consultation of a Pulmonary Rehabilitation Programme Data Set, doi: 10.6084/m9.figshare.928540 ^31^

## Consent

 All participants provided written informed consent.
