# Supplementary material for: Nominal Group Technique consultation of a Pulmonary Rehabilitation Programme
Source: F1000Res. 2014 Feb 13;3:42. [Version 1] doi: 10.12688/f1000research.3-42.v1 (PMC4103494; doi:10.12688/f1000research.3-42.v1)
Supplement: Peer review history

# Peer review: Referee response for version 1

**DOI:** 10.5256/f1000research.3790.r5251

**Published:** 2014-06-26

**Authors:** Amanda Stears

**Affiliations:** 1 Department of Physiotherapy, School of Health Sciences, Queen Margaret University, Edinburgh, Scotland, UK

The article gives a clear introduction to the concept of Pulmonary Rehabilitation (PR) acknowledging the evidence supporting the intervention and the positive impact that is has on participants lives post programme. Most importantly, it seeks to gather the opinions of “significant others” and professionals within the clinical team, something that is often overlooked but that could be a key component in service development.

There is a clear introduction to the use of the Nominal Group Technique and its usefulness in facilitating all participants’ views to be gathered and the aim of providing a rank of perceived benefits and challenges in pulmonary rehabilitation is clearly stated.     

The authors have used the Nominal Group Technique successfully to provide a useful ranking of themes and challenges from the perspectives of patients, professionals and significant others. These are useful to challenge current assumptions within Pulmonary Rehabilitation Programmes, but the study would benefit from consideration of the socio-demographics of the group as these also impact on health related outcomes. The small sample size also makes it difficult to generalise to a wider population, something which the authors themselves acknowledge. There is also a strong male bias in the sample group, what affect did this have on the results?

The data and techniques used are clearly described and well presented and are representative and reflective of findings in the clinical setting.

In conclusion, going forwards further research is required to establish whether the rankings are concurrent across the country, and if so do they have a role in guiding PR and assisting patients and significant others in overcoming the perceived barriers. Many of the themes and issues identified are already known to those working within the clinical setting and are addressed through education and management within PR in the UK already. There are also assumptions in practice that patients “Learn” as is stated on page 6 in the Thematic Template generation. How is this evidenced? One finding that the authors also highlight is that significant others have more time for themselves as patients become more independent therefore supporting the self management of PR; this would benefit from further exploration.

I have read this submission. I believe that I have an appropriate level of expertise to confirm that it is of an acceptable scientific standard.

# Peer review: Referee response for version 1

**DOI:** 10.5256/f1000research.3790.r4411

**Published:** 2014-04-08

**Authors:** Pat G. Camp, Carmen Sima

**Affiliations:** 1 Department of Physical Therapy, University of British Columbia, Vancouver, BC, Canada

**STUDY PURPOSE**

The purpose of the present study was to describe the perceived benefits and challenges of pulmonary rehabilitation program for patients with COPD from the point of view of patients, professionals, and significant others (carers).

**TITLE AND ABSTRACT**

The title reflects the content of the article. The abstract provides a suitable summary of the work.  The conclusion would be strengthened by a main message.

**INTRODUCTION**

The introduction provides detailed information about the area of research, gaps in the literature, and techniques employed to answer the research question. The references to the relevant literature on the mixed method design were also provided in order to support the proposed methodology.

**METHODS**

The design of the study was also described clearly, including thematic generation and consensus, as well as responsibilities for integration. However, the authors state that the focus is on the quantitative analysis rather than the qualitative analysis, yet the analysis described appears to be qualitative in nature, and the authors comment on the need to maintain qualitative rigour throughout the study. A ranking by median scores by the participants is still qualitative in nature.  If the intent is to undertake a quantitative analysis of the data, the selection of the median rank and interquartile ranges as appropriate statistical tests that is meant to answer the study research question seems insufficient. The authors might want to explore the differences between the different groups in regard to the perceived benefits and challenges of pulmonary rehabilitation program for patients with COPD. We consider that there is a need for clearly stating the hypothesis being tested and a motivation for selecting specific statistical analysis, should a quantitative analysis be intended. The individual data table would be better presented in the results section.

**RESULTS**

The results section is appropriately explained, but the emphasis is again placed on the qualitative than quantitative research findings. In addition, the authors state that 20 participants were initially included in the study (8 patients, 8 professionals and 4 others) and 12 participants were finally included in the analysis. Given the small sample size, there is also a need to specify the status of the participants who did not complete the study. This information may influence the interpretation of the study results. There is also a disagreement between the evaluable responses across the paper: 60% on the page 6 versus 70% on the page 9. The authors also state that COPD patients who were currently participating in or completed a pulmonary rehabilitation program within the last 2 years were approached to participate in the study. However, Table 1 shows 2005 as year of PR program for one COPD patient. It is also worth mentioning the weak participation of physiotherapists in this study, although the pulmonary rehabilitation program was the main component of this research and the physiotherapists play a significant role in the delivery of pulmonary rehabilitation program. The thematic template generation does not offer clear guidance to the reader on what are the clear benefits and challenges – the list is somewhat self-evident and simplistic, and appears to focus on the benefits of the pulmonary rehabilitation program, but the challenges are not clearly articulated.

**DISCUSSION**

The conclusions are generally balanced and justified, although special emphasis is placed on the qualitative findings. Some of the discussion would be better suited to the results. The main benefits and challenges of pulmonary rehabilitation for patients, carers, and professionals as presented in the themes are not specific enough to be of use for making recommendations for improving the pulmonary rehabilitation program.

We have read this submission. We believe that we have an appropriate level of expertise to confirm that it is of an acceptable scientific standard, however we have significant reservations, as outlined above.

# Peer review: Referee response for version 1

**DOI:** 10.5256/f1000research.3790.r3648

**Published:** 2014-03-05

**Authors:** Kate Bullen

**Affiliations:** 1 Department of Psychology, Aberystwyth University, Wales, UK

This is an interesting and useful article that explores the implications and lived experience of people dealing with the challenges of a debilitating chronic disease (COPD). This is not a "sexy" area of health care research but it is an important one as COPD substantially limits life in both quantity and quality. As such it is a worthy area of investigation, as the aim of this article is to identify how to improve the delivery of service for a sometimes overlooked patient group. 

The article is well presented and reflects a high level of attention to detail in design and analysis. The research team has identified an appropriate method of investigation which is sufficiently novel to generate new knowledge that will potentially inform clinical practice. Details of the data collection and analysis processes are well presented and the data are discussed comprehensively. The research team acknowledges that there are limitations in the study in terms of the level of participants, and the level of geographical specificity of the study. Whilst this is a sensible approach such caveats should not detract from the potential value of the study for practitioners who deliver such programmers. Finally, the conclusions are well balanced and insightful with a clear pathway to future research identified.

I have read this submission. I believe that I have an appropriate level of expertise to confirm that it is of an acceptable scientific standard.
